# Supplementary material for: Cell-specific exon methylation and CTCF binding in neurons regulate calcium ion channel splicing and function
Source: eLife. 2020 Mar 26;9:e54879. doi: 10.7554/eLife.54879 (PMC7124252; doi:10.7554/eLife.54879)
Supplement: Supplementary file 1. [file elife-54879-supp1.docx]

| **Key Resources Table** | | | | |
| --- | --- | --- | --- | --- |
| **Reagent type or resource** | **Designation** | **Source or reference** | **Identifiers** | **Additional information** |
| Genetic reagent (*Mus musculus, females and males*) | *Trpv1^Cre^* mouse | The Jackson Laboratory | JAX:017769; RRID:IMSR_JAX:017769 |  |
| Genetic reagent (*Mus musculus, females and males*) | *lox-STOP-lox^ChR2-EYFP^* mouse | The Jackson Laboratory | JAX:012569; RRID:IMSR_JAX:012569 |  |
| Genetic reagent (*Mus musculus, females and males*) | *lox-STOP-lox^TdTomato^* mouse | The Jackson Laboratory | JAX:007908; RRID:IMSR_JAX:007908 |  |
| Genetic reagent (*Escherichia coli*) | One Shot™ TOP10 Electrocomp™ *E. coli* | Thermo Fisher Scientific | Thermo Fisher Scientific:C404050 |  |
| Genetic reagent (*Escherichia coli*) | One Shot™ Stbl3™ Chemically Competent *E. coli* | Thermo Fisher Scientific | Thermo Fisher Scientific:C737303 |  |
| Cell line  (*Rattus norvegicus*/ *Mus musculus*) | F11 cells | PMID: 29067356 | ECACC:08062601 | Somatic cell hybrid of a rat embryonic dorsal root ganglion and mouse neuroblastoma cell line N18TG2.  Gift from Dr. Probal Banerjee |
| Antibody | Rabbit monoclonal anti-CTCF | Cell Signaling Technology | Cell Signaling Technology:3418; RRID:AB_2086791 | (1:1000); see ChIP protocol |
| Antibody | Rabbit monoclonal anti-GAPDH | Cell Signaling Technology | Cell Signaling Technology:2118; RRID:AB_561053 | (1:1000); see Western Blot protocol |
| Antibody | Normal rabbit IgG | Cell Signaling Technology | Cell Signaling Technology:2729: RRID:AB_1031062 | See ChIP protocol |
| Antibody | Mouse monoclonal anti-CTCF | BD Biosciences | Cell Signaling Technology:612148; RRID:AB_399519 | See EMSA protocol |
| Antibody | Horseradish peroxidase-labeled anti–rabbit secondary antibody | Kirkegaard & Perry Laboratories | Kirkegaard & Perry Laboratories:474-1516 | (1:15,000); see Western Blot protocol |
| Recombinant DNA reagent | cDNA encoding *Tet1* | Addgene | Addgene:60938; RRID:Addgene_60938 |  |
| Recombinant DNA reagent | cDNA encoding *Tet2* | Addgene | Addgene:60939; RRID:Addgene_60939 |  |
| Recombinant DNA reagent | cDNA encoding *Tet3* | Addgene | Addgene:60940; RRID:Addgene_60940 |  |
| Recombinant DNA reagent | *dCas9-Tet1* | Addgene | Addgene:84475; RRID:Addgene_84475 |  |
| Recombinant DNA reagent | gRNA | Addgene | Addgene:84477; RRID:Addgene_84477 |  |
| Recombinant DNA reagent | recombinant CTCF tagged with GFP | PMID:  16107875 |  |  |
| Recombinant DNA reagent | GFP | PMID:  16107875 |  |  |
| Sequence-based reagent | Control siRNA | GE Healthcare Dharmacon | GE Healthcare Dharmacon:D-001206-13 |  |
| Sequence-based reagent | *Dnmt1* siRNA | GE Healthcare Dharmacon | GE Healthcare Dharmacon:M-056796-01 |  |
| Sequence-based reagent | *Dnmt3a* siRNA | GE Healthcare Dharmacon | GE Healthcare Dharmacon:M-065433-01 |  |
| Sequence-based reagent | *Dnmt3b* siRNA | GE Healthcare Dharmacon | GE Healthcare Dharmacon:M-044164-01 |  |
| Sequence-based reagent | *Ctcf* siRNA | GE Healthcare Dharmacon | GE Healthcare Dharmacon:M-044693-01 |  |
| Sequence-based reagent | Fw-e37a | This paper | PCR primers | ACCTGTAACATTTCCTTTCCAG |
| Sequence-based reagent | Rv-e37a | This paper | PCR primers | GAGGCTCTGAAGTTGCAAAC |
| Sequence-based reagent | Fw-e37b | This paper | PCR primers | CCTCTGGAACGGGTTTCCAG |
| Sequence-based reagent | Rv-e37b | This paper | PCR primers | TCAGTGCAGGGTCAAGGTCTAC |
| Sequence-based reagent | JLS19 | This paper | PCR primers | TTGTTGCTGTAATCATGGACAA |
| Sequence-based reagent | JLS20 | This paper | PCR primers | CAGCCCAGACTCGAATGAAT |
| Sequence-based reagent | JLS09 | This paper | PCR primers | CGCAATACAACGCAACAAAC |
| Sequence-based reagent | JLS10 | This paper | PCR primers | GAGGTGGGGACATGTGTTTC |
| Sequence-based reagent | JLS21 | PMID: 24698270 | PCR primers | AATGTGTCCGTCGTGGATCT |
| Sequence-based reagent | JSL22 | PMID: 24698271 | PCR primers | GTTGAAGTCGCAGGAGACAA |
| Sequence-based reagent | JLS47 | This paper | PCR primers | TATTTTTTATTGTAGATTGGGTGGG |
| Sequence-based reagent | JLS48 | This paper | PCR primers | TCAAATAAAAACTCTAAAATTACAAA |
| Sequence-based reagent | JLS53 | Universal primer | PCR primers | CGACTCACTATAGGGAGAGCGGC |
| Sequence-based reagent | JLS54 | Universal primer | PCR primers | AAGAACATCGATTTTCCATGGCAG |
| Sequence-based reagent | JLS65 | This paper | PCR primers | GAAACTCACCCTAACTG |
| Sequence-based reagent | JLS59 | This paper | PCR primers | TTGGACCTTGTAGGCCAACCTACG |
| Sequence-based reagent | JLS60 | This paper | PCR primers | AAACCGTAGGTTGGCCTACAAGGT |
| Sequence-based reagent | JLS61 | This paper | PCR primers | TTGGCAGTTGCCGGATTCATTATA |
| Sequence-based reagent | JLS62 | This paper | PCR primers | AAACTATAATGAATCCGGCAACTG |
| Sequence-based reagent | JLS63 | This paper | PCR primers | TTGGCCCCGGGGAAAAATTTTTTT |
| Sequence-based reagent | JLS64 | This paper | PCR primers | AAACAAAAAAATTTTTCCCCGGGG |
| Peptide, recombinant protein | CTCF human recombinant protein | Abnova | Abnova:H00010664-P01 |  |
| Peptide, recombinant protein | Collagenase | Sigma-Aldrich | Sigma-Aldrich:C9891 |  |
| Peptide, recombinant protein | Trypsin | Sigma-Aldrich | Sigma-Aldrich:85450C |  |
| Peptide, recombinant protein | Precision Plus Protein™ Dual Color Standards | Bio-Rad | Bio-Rad:1610374 |  |
| Peptide, recombinant protein | Precision Protein™ StrepTactin-HRP Conjugate | Bio-Rad | Bio-Rad:1610381 |  |
| Peptide, recombinant protein | EpiMark® Hot Start Taq DNA Polymerase and reaction buffer | New England Biolabs | New England Biolabs:M0490S |  |
| Peptide, recombinant protein | RNase Cocktail™ Enzyme Mix | Invitrogen | Invitrogen:AM2286 |  |
| Peptide, recombinant protein | Proteinase K | Ambion | Ambion:AM2546 |  |
| Peptide, recombinant protein | AarI restriction enzime | Thermo Fisher Scientific | Thermo Fisher Scientific:ER1581 |  |
| Peptide, recombinant protein | Q5® High-Fidelity DNA Polymerase and reaction buffer | New England Biolabs | New England Biolabs:M0491 |  |
| Peptide, recombinant protein | T4 DNA Ligase and buffer | New England Biolabs | New England Biolabs:M0202S |  |
| Commercial assay or kit | Pierce™ BCA Protein Assay Kit | Thermo Fisher Scientific | Thermo Fisher Scientific:23227 |  |
| Commercial assay or kit | QIAamp DNA Mini Kit | QIAGEN | QIAGEN:51304 |  |
| Commercial assay or kit | EpiTect Bisulfite Kit | QIAGEN | QIAGEN:59104 |  |
| Commercial assay or kit | QIAquick gel extraction kit | QIAGEN | QIAGEN:28704 |  |
| Commercial assay or kit | CloneJET PCR Cloning Kit | Thermo Fisher Scientific | Thermo Fisher Scientific:K1232 |  |
| Commercial assay or kit | QIAprep Spin Miniprep Kit | QIAGEN | QIAGEN:27106 |  |
| Commercial assay or kit | QIAquick PCR Purification Kit | QIAGEN | QIAGEN:28106 |  |
| Commercial assay or kit | Pierce™ Biotin 3' End DNA Labeling Kit | Thermo Fisher Scientific | Thermo Fisher Scientific:89818 |  |
| Commercial assay or kit | LightShift Chemiluminescent EMSA Kit | Thermo Fisher Scientific | Thermo Fisher Scientific:20148 |  |
| Commercial assay or kit | SuperScript® III First-Strand Synthesis System with Poli-dT primers | Invitrogen | Invitrogen:  18080051 |  |
| Commercial assay or kit | MethylFlash Methylated DNA 5-mC Quantification Kit | EpiGentek | EpiGentek:P-1030 |  |
| Commercial assay or kit | MethylFlash Global DNA Hydroxymethylation 5-hmC ELISA Easy Kit | EpiGentek | EpiGentek:P-1032 |  |
| Chemical compound, drug | 5-Azacytidine (5-Aza) | Sigma-Aldrich | Sigma-Aldrich:A2385 |  |
| Chemical compound, drug | 2-hydroxyglutarate (2-HG) | TRC Toronto Research Chemicals | TRC Toronto Research Chemicals:  H942596 |  |
| Chemical compound, drug | Lipofectamine™ 2000 Transfection Reagent | Invitrogen | Invitrogen:  11668027 |  |
| Software, algorithm | MethPrimer | PMID: 12424112 | RRID:SCR_010269 | URL: http://urogene.org/ |
| Software, algorithm | Primer3 | PMID: 17379693 | RRID:SCR_003139 | URL: http://primer3.ut.ee |
| Software, algorithm | ImageJ | NIH | RRID:SCR_003070 | URL: https://imagej.net/ |
| Software, algorithm | Prism 8 | GraphPad | RRID:SCR_005375 | URL: http://graphpad.com/scientific-software/prism/ |
| Other | Isoflurane | Patterson Veterinary | Patterson Veterinary:  14043070406 |  |
| Other | Isopropyl alcohol | Dynarex | Dynarex:1113 |  |
| Other | Povidone-iodine solution | Dynarex | Dynarex:1108 |  |
| Other | HBSS | Gibco | Gibco:24020117 |  |
| Other | PBS | Gibco | Gibco:10010031 |  |
| Other | Fetal Bovine Serum | Gibco | Gibco:A3160601 |  |
| Other | TRIzol® LS Reagent | Invitrogen | Invitrogen:  10296010 |  |
| Other | Dulbecco’s modified Eagle’s medium | Gibco | Gibco:10569010 |  |
| Other | Opti-MEM™ reduced serum medium | Gibco | Gibco:31985070 |  |
| Other | cOmplete™, Mini, EDTA-free Protease Inhibitor Cocktail | Roche | Roche:  04693159001 |  |
| Other | ProSignal® Dura ECL Reagent | Genesee Scientific | Genesee Scientific:20-301 |  |
| Other | SeaKem® LE agarose | Lonza | Lonza:50004 |  |
| Other | Magna ChIP™ Protein A+G Magnetic Beads | Millipore | Millipore:16-663 |  |
| Other | TRIzol | Invitrogen | Invitrogen:  15596018 |  |
| Other | NEBuffer™ 3.1 | New England Biolabs | New England Biolabs:B7203S |  |
| Other | Sterile 6-0 coated vicryl suture | Ethicon | Ethicon:J833G |  |
| Other | Deltaphase® Isothermal Pads | Braintree scientific | Braintree scientific:DPIP |  |
| Other | Plantar Analgesia Meter | IITC | IITC:II-390G |  |
| Other | Flowmi® Cell Strainer | Sigma-Aldrich | Sigma-Aldrich:  BAH136800070 |  |
| Other | Amersham Protan 0,45 mM NC, nitrocellulose membrane | GH Healthcare Life Science | GH Healthcare Life Science:10600002 |  |
| Other | Amersham™ Hybond™-N+ 0.45 µm nitrocellulose membrane | GH Healthcare Life Science | GH Healthcare Life Science:95038-376 |  |
