# Supplementary material for: Cell-specific exon methylation and CTCF binding in neurons regulate calcium ion channel splicing and function
Source: eLife. 2020 Mar 26;9:e54879. doi: 10.7554/eLife.54879 (PMC7124252; doi:10.7554/eLife.54879)
Supplement: Transparent reporting form [file elife-54879-transrepform.pdf]

eLIFE
1st Floor
24 Hills Road
Cambridge CB2 1JP, UK
P 01223 855340
W elifesciences.org
T @elife

eLife's transparent reporting form

We encourage authors to provide detailed information *within their submission* to facilitate the interpretation and replication of experiments. Authors can upload supporting documentation to indicate the use of appropriate reporting guidelines for health-related research (see EQUATOR Network), life science research (see the BioSharing Information Resource), or the ARRIVE guidelines for reporting work involving animal research. Where applicable, authors should refer to any relevant reporting standards documents in this form.

If you have any questions, please consult our Journal Policies and/or contact us: editorial@elifesciences.org.

Sample-size estimation

- You should state whether an appropriate sample size was computed when the study was being designed
- You should state the statistical method of sample size computation and any required assumptions
- If no explicit power analysis was used, you should describe how you decided what sample (replicate) size (number) to use

Please outline where this information can be found within the submission (e.g., sections or figure legends), or explain why this information doesn't apply to your submission:

No power analysis was used to pre-determine sample size during study design. Instead, sample size was empirically determined based on our experience knowing the biological and technical variability and on previously published studies in our field for qPCR, bisulfite sequencing and behavioral assessment of paw sensitivity using the Hargreaves test.

Sample size for each experimental condition is reported in figure legends and/or in the Materials and Methods section.

Replicates

- You should report how often each experiment was performed
- You should include a definition of biological versus technical replication
- The data obtained should be provided and sufficient information should be provided to indicate the number of independent biological and/or technical replicates
- If you encountered any outliers, you should describe how these were handled
- Criteria for exclusion/inclusion of data should be clearly stated
- High-throughput sequence data should be uploaded before submission, with a private link for reviewers provided (these are available from both GEO and ArrayExpress)

eLife Sciences Publications, Ltd is a limited liability non-profit non-stock corporation incorporated in the State of Delaware, USA, with company number 5030732, and is registered in the UK with company number FC030576 and branch number BR015634 at the address 1st Floor, 24 Hills Road, Cambridge CB2 1JP | August 2014

Please outline where this information can be found within the submission (e.g., sections or figure legends), or explain why this information doesn't apply to your submission:

For each experiment, we report the number of biological replicates performed. These are found in corresponding figure legends and/or in the results section. The meaning of the replicates reported are defined in the last paragraph of each figure legend.

In the spared nerve injury model, we set an exclusion criterion for the development of thermal hyperalgesia 7 days post-surgery as at least a 20% reduction in paw withdrawal latency compared to pre-surgery values. All animals met the criteria, none were excluded.

No data were excluded from our analyses, and we did not perform analyses to identify or exclude outliers.

Statistical reporting

- Statistical analysis methods should be described and justified
- Raw data should be presented in figures whenever informative to do so (typically when N per group is less than 10)
- For each experiment, you should identify the statistical tests used, exact values of N, definitions of center, methods of multiple test correction, and dispersion and precision measures (e.g., mean, median, SD, SEM, confidence intervals; and, for the major substantive results, a measure of effect size (e.g., Pearson's r, Cohen's d)
- Report exact p-values wherever possible alongside the summary statistics and 95% confidence intervals. These should be reported for all key questions and not only when the p-value is less than 0.05.

Please outline where this information can be found within the submission (e.g., sections or figure legends), or explain why this information doesn't apply to your submission:

Details regarding statistical analyses are included in the corresponding figure legends as well as in the Statistical Analysis subheading in the Materials and Methods section. All comparisons, sample size (total number of biological replicates), mean and SEM per condition and statistical tests are reported. We report exact p-values in the figure legends.

Individual data points are plotted throughout.

For CTCF protein quantification, images of complete Western blots are shown.

(For large datasets, or papers with a very large number of statistical tests, you may upload a single table file with tests, Ns, etc., with reference to sections in the manuscript.)

Group allocation

- Indicate how samples were allocated into experimental groups (in the case of clinical studies, please specify allocation to treatment method); if randomization was used, please also state if restricted randomization was applied
- Indicate if masking was used during group allocation, data collection and/or data analysis

Please outline where this information can be found within the submission (e.g., sections or figure legends), or explain why this information doesn't apply to your submission:

For both *in vitro* and *in vivo* experiments control and treatment groups were run same experimental day. Treatments on cultured cells were randomized over the course of each experiment as described in the methods section.

Specifically, for *in vivo* experiments, mice were assigned to two groups, Spared Nerve Injury and Sham, procedures randomized and the experimenter was masked to surgery type to assess behavior and analyses. Details of randomization, testing, and masking are described in the Thermo-sensitivity Assessment subheading in the Materials and Methods section.

No clinical studies were performed.

Additional data files ("source data")

- We encourage you to upload relevant additional data files, such as numerical data that are represented as a graph in a figure, or as a summary table
- Where provided, these should be in the most useful format, and they can be uploaded as "Source data" files linked to a main figure or table
- Include model definition files including the full list of parameters used
- Include code used for data analysis (e.g., R, MatLab)
- Avoid stating that data files are "available upon request"

Please indicate the figures or tables for which source data files have been provided:

We used the ENCODE database and UCSC browser to retrieve and display CTCF-ChIP-seq and conservation tracks. We included links in the corresponding figure legends that direct the reader to data visualization shown in Figure 1 and in Supplemental Figure 1.
